# Supplementary material for: Capacity optimization configuration and multi-dimensional value evaluation of integrated energy system with power-to-hydrogen
Source: PLoS One. 2025 Apr 17;20(4):e0320486. doi: 10.1371/journal.pone.0320486 (PMC12005829; doi:10.1371/journal.pone.0320486)
Supplement: Attachment — Submitted filename: Response to Reviewers.docx [file pone.0320486.s002.docx]

RESPONSES TO REVIEWERS

We would like to thank the reviewers and editor for their thoughtful comments and suggestions, which certainly help us to improve the quality of this paper. Specific as well as general changes are made according to the reviewers' comments, and are specifically ***highlighted in red in the revised manuscript****.* Below is a detailed explanation of the changes made to address all the concerns.

# RESPONSES TO Reviewer 1

*1) The paper uses the Analytic Hierarchy Process (AHP) to determine the weight coefficients among different objectives. How can the issue of excessive conservatism be mitigated? Please provide additional explanations.*

**Response:** Thank you very much for your valuable feedback regarding the **conservatism issue** associated with the **Analytic Hierarchy Process (AHP)**. In response to your suggestion, AHP can sometimes lead to overly conservative weight assignments, especially when subjective judgments are involved. To mitigate this issue, we incorporated **sensitivity analysis** into the weight coefficient determination process. Please refer to the revised manuscript, where the changes are highlighted in red font in Section 5.2.2 on page 19. The detailed modifications are as follows:

(2) Weight coefficients between different objectives

The determination of the weight coefficients $a_{1},a_{2},a_{3},a_{4}$ in Formula (1) is based on the importance judgment matrix in Table 2, calculated using the AHP method, and then derived through sensitivity analysis. As shown in Figure 8, the analysis results indicate that the system achieves optimal performance $a_{1}=0.3706,a_{2}=0.1433,a_{3}=0.2813,a_{4}=0.2048$ .

(a) Weight coefficient of a1	(b) Weight coefficient of a2

*2) As stated in the paper, nonlinear problems are typically transformed into convex problems to achieve accurate global optimal solutions. In this context, the work “enabling high-efficiency economic dispatch of hybrid ac/dc networked microgrids: steady-state convex bi-directional converter models” should be discussed and cited in the literature review to enhance the quality of the paper.*

**Response:** Your suggestion is highly valuable to us. In response to your comment, we have carefully reviewed the work you referred to and agree that it offers important insights into transforming nonlinear problems into convex ones. As recommended, we have added a discussion of this work to Section 1.1 on page 2 to further enhance the context of our study. Please refer to the revised manuscript, where the changes are highlighted in red font . The detailed modifications are as follows:

Reference [14] proposes a least squares approximation method that simplifies the complex trigonometric functions representing the conversion efficiency of the bidirectional converter (BDC) as it varies with power, transforming the original non-convex relationship into a computationally efficient convex form.

Reference：

[14] Liang Z, Chung C Y, Zhang W, Wang Q, Li W, and Wang C. Enabling high-efficiency economic dispatch of hybrid AC/DC networked microgrids: Steady-state convex bi-directional converter models[J]. IEEE Transactions on Smart Grid, 2024, 16(1): 45-61. doi: 10.1109/TSG.2024.3454050

*3) Why not use more advanced Pareto-based methods to handle the multi-objective optimization problem?* *Please provide further explanations.*

**Response:** Thank you for the valuable suggestion. Regarding the use of Pareto-based methods for handling multi-objective optimization problems, we opted for the weight-based approach using the **Analytic Hierarchy Process (AHP)** for the following reason. In multi-objective optimization problems, **Pareto-based methods** typically require trade-offs among objectives and the generation of non-dominated solution sets, which significantly increases **computational complexity.** Given that the problem studied in this paper involves multiple constraints and high computational demands, we adopted the **AHP method** to aggregate the four objectives into a single objective, thereby simplifying the model and improving **computational efficiency.** In future work, we plan to introduce more advanced **Pareto-based methods** to extend the optimization problem, exploring the potential applications and advantages of multi-objective optimization methods in this field. In response to your suggestion, we have provided further explanations in the revised manuscript in Section 1.2 (Research Gap and Contributions) on page 5, to explain why we opted for a weight-based approach rather than more advanced Pareto-based methods. Please refer to the revised manuscript, where the changes are highlighted in red font. The detailed modifications are as follows:

Meanwhile, in solving the P2H equipment capacity optimization configuration model, traditional mathematical solution methods, due to the higher-order nonlinearity of the objective function in integrated energy systems, have become less applicable. The use of metaheuristic algorithms can effectively address this issue. Furthermore, in multi-objective optimization problems, methods based on Pareto usually require trade-offs between objectives and generate non-dominated solution sets, which significantly increase computational complexity. However, by combining multiple objectives into a single objective using weight coefficients, the model can be effectively simplified, thus improving computational efficiency.

*4) Integrated energy systems usually involve a high proportion of renewable energy, and renewable energy generation is inherently uncertain. A discussion on this aspect should be added to the paper, particularly regarding robust optimization methods, such as “an iteration-free hierarchical method for the energy management of multiple-microgrid systems with renewable energy sources and electric vehicles.” It is recommended that the authors elaborate on this and include the relevant references.*

**Response:** Thank you very much for your valuable suggestion. Regarding the issue of the high proportion of renewable energy in integrated energy systems and the **inherent uncertainty of renewable energy generation**, your comment has provided us with a highly valuable research direction. This paper currently focuses on optimization problems under **deterministic conditions** and does not address the **uncertainty** of renewable energy. The **robust optimization method** you recommended (such as “an iteration-free hierarchical method for the energy management of multiple-microgrid systems with renewable energy sources and electric vehicles”) offers important insights, especially in dealing with **uncertainties** related to renewable energy and electric vehicles.

In future research, we plan to explore the **application of robust optimization methods** in integrated energy systems from the perspective of **uncertainty** and conduct experiments and validations. The current study lays the **foundation** for subsequent work, and the detailed discussion on **robust optimization methods** will be a **key focus** of our next steps. Once again, thank you for your suggestion, which provides significant guidance for further improving our research.

*5)* *What is the motivation for using a meta-heuristic algorithm to solve the model rather than established solvers like CPLEX or Gurobi? Please clarify this choice.*

**Response:** Thank you very much for your valuable suggestions. The reason for selecting metaheuristic algorithms for solving the power-to-hydrogen (P2H) capacity optimization configuration model is due to the **high nonlinearity** of the model. **Gurobi or CPLEX** cannot directly solve this model, but by using **metaheuristic algorithms**, we can avoid the nonlinearity issues and simplify the solution process. We have also provided further explanation of this issue in Section 1.2 on page 5, which discusses **the research gap and contributions**. For specific details on the modifications, please refer to Modification Comment 3.

*6) Regarding Table 5, how can the results obtained by the proposed algorithm be demonstrated to outperform those of other algorithms? Please provide additional explanations.*

**Response:** Thank you very much for your valuable feedback regarding Table 5. To further demonstrate the superiority of the proposed IBBPSO algorithm over other algorithms, we have added two new algorithms, GWO and WOA, to the table for comparison, and we have rewritten the conclusion. Please refer to section 5.3.1 on page 21 of the revised paper. Additionally, in section 5.3.2 (pages 21-23), we further demonstrate that the IBBPSO algorithm outperforms other algorithms through convergence curve plots, and multi-factor ANOVA plots. Please refer to the revised manuscript, where the changes are highlighted in red font. The detailed modifications are as follows:

Table 7. Numerical results under different algorithms

The numerical results in Table 7 highlight the superior performance of the proposed IBBPSO algorithm compared to other algorithms. Specifically, the IBBPSO algorithm achieves the highest comprehensive benefits of 376.447, which is 11.09% and 9.8% higher than the BBPSO and PSO algorithms, respectively. Compared to the GWO and WOA algorithms, the comprehensive benefits V of the IBBPSO algorithm are 33.57% and 17.7% higher, respectively, demonstrating its excellent optimization capability across multiple objectives. In terms of flexibility benefits , the IBBPSO algorithm achieves a value of 296.229, which is 14.79% and 9.21% higher than BBPSO and PSO, respectively, and 41.87% and 54.74% higher than GWO and WOA, respectively. Furthermore, the IBBPSO algorithm demonstrates significant advantages in renewable energy consumption benefits and environmental benefits , highlighting its effectiveness in balancing economic, environmental, and flexibility objectives.

5.3.2 Algorithm Comparison Analysis

Fig 9 shows the convergence curve comparison diagram of the algorithm—the significant advantages of the IBBPSO algorithm in convergence performance. Compared with the PSO, BBPSO, GWO and WOA, the convergence speed is faster, and the convergence result is better. The ' multi-factor ANOVA ' is used to analyze the comprehensive benefit results with a 95 % confidence interval to compare the influence of different algorithms on solving the optimization model. The results are shown in Fig 10. The comprehensive benefit obtained by the IBBPSO algorithm is significantly higher than that of the other two algorithms, which proves its superior optimization ability.

Fig 9. Algorithm convergence curve

Fig 10. Comparison of system benefits of different algorithms

# RESPONSES TO REVIEWER 2

*1) Please include the main numerical results in both the Abstract and Conclusion sections to summarize key findings clearly.*

**Response:** Thank you for your constructive suggestion. In response to your comment, we have revised both the Abstract and Conclusion sections to include the main numerical results, clearly summarizing the key findings. Please refer to the text in red font on page 1and page 28 of the revised manuscript. The details are as follows:

Abstract:

The experimental results show that the IBBPSO algorithm exhibits better performance in solving the optimization model. Compared to PSO, IBBPSO, GWO, and WOA algorithms, it improves by 9.8%, 11.09%, 33.57%, and 17.7%, respectively. The optimal solution is achieved when the P2H equipment is configured to 50 MW.

Conclusion:

(1) The IBBPSO algorithm introduced global and local search factors to expand the search range, prevent the search results from falling into local optimum, and significantly enhance the algorithm's optimization ability. When solving the optimization configuration model, the IBBPSO algorithm yields a comprehensive benefit of 376.447. Compared to PSO, IBBPSO, GWO, and WOA algorithms, it improves by 9.8%, 11.09%, 33.57%, and 17.7%, respectively.

(2) The constructed multi-dimensional value evaluation system uses the combined weight of the AHP-entropy weight method to avoid the mutual interference between evaluation indicators and successfully identifies the reasonable configuration of P2H equipment in the IES when the comprehensive benefits brought by the P2H equipment to the system are the largest. The experiment shows that the highest comprehensive value is achieved when the capacity of the P2H equipment is 50 MW.

*2) Compare the work presented in this paper with other studies in the literature, preferably in a tabular format, to highlight differences and improvements.*

**Response:** Thank you for your valuable suggestion. In response to your comment, we have included a detailed comparison with recent relevant studies in the literature in a tabular format to clearly highlight the differences and improvements of our work. The newly added Table 1 provides a comprehensive review of key studies, focusing on optimization configuration models, objective functions, and solution methods. Please see the red font in Section 1.1 on page 4 of the revised manuscript. The details are as follows :

Table 1 Review of recent relevant studies along with the present study

*3) The introduction needs to be enhanced by discussing more recent meta-heuristic algorithms. Consider referencing studies such as:* https://doi.org/10.1016/j.asej.2022.102092; https://doi.org/10.1016/j.egyr.2022.04.066; https://doi.org/10.1038/s41598-024-76410-0

**Response:** Thank you for your valuable suggestion. In response to your comment, we have revised the Introduction section to incorporate a discussion on recent developments in meta-heuristic algorithms. The referenced studies have been reviewed and cited to enhance the relevance and comprehensiveness of the introduction. Please see the red font in Section 1.1 on page 3 of the revised manuscript. The details are as follows :

Reference [16] proposes a hybrid Runge Kutta-gradient-based optimization algorithm, which combines the Runge Kutta optimizer and a gradient-based optimizer to solve a generation and transmission expansion planning model embedded with energy storage systems. Reference [17] develops a hybrid artificial rabbits sine-cosine algorithm to address complex non-convex problems in AC optimal power flow models. Reference [19] combines the features of the Marine Predators Algorithm (MPA) and the Honey Badger Algorithm (HBA) to propose a hybrid algorithm (MPA-HBA) for solving the integrated hosting capacity model for electric vehicles.

Reference：

[16] Rawa M, AlKubaisy Z M, Alghamdi S, Refaat M M, Ali Z M, and Aleem S H A. A techno-economic planning model for integrated generation and transmission expansion in modern power systems with renewables and energy storage using hybrid Runge Kutta-gradient-based optimization algorithm[J]. Energy Reports, 2022, 8: 6457-6479. doi: 10.1016/j.egyr.2022.04.066
[17] Vellingiri M, et al. Maximum hosting capacity estimation for renewables in power grids considering energy storage and transmission lines expansion using hybrid sine cosine artificial rabbits algorithm[J]. Ain Shams Engineering Journal, 2023, 14(5): 102092. doi: 10.1016/j.asej.2022.102092
[18] Al-Dhaifallah M, Refaat M M, Alaas Z, Abdel Aleem S H, and Ali Z M. Enhancing hosting capacity for electric vehicles in modern power networks using improved hybrid optimization approaches with environmental sustainability considerations[J]. Scientific Reports, 2024, 14(1): 25607.doi: 10.1038/s41598-024-76410-0

*4) Provide a detailed explanation of all algorithm parameters. Clarify why and how specific values were chosen and discuss how varying these values impacts the algorithm's overall performance.*

**Response:** Thank you for your constructive suggestion. To address your comment, we have provided a detailed explanation of the key algorithm parameters and their settings in the revised manuscript. Specifically, the parameters for the IBBPSO algorithm include **population size (Popsize)**, **number of iterations (Iteration)**, and **penalty factor (r)**. To determine the optimal parameter values, we employed the **Taguchi method** and designed an $\text{L}_{16}(3^{3})$-orthogonal experimental table, as shown in Table 5 . Please see the red font in Section 5.2.2 on pages 18-19 of the revised manuscript. The details are as follows :

5.2.2 Parameter settings

(1) Algorithm parameters

The key algorithm parameters that need to be set for the IBBPSO algorithm include population size (Popsize), number of iterations (Iteration), and penalty factor (r). The Taguchi method is used to examine the impact of different levels of algorithm parameters on the solution. Each parameter is set to three levels, and an $\text{L}_{16}(3^{3})$-orthogonal experimental table is established, as shown in Table 5. Each experimental group runs 10 times, and the overall evaluation normalized value method is used to calculate the total evaluation normalized value (GM) to assess the parameters. The calculation formula is as follows:

 (32)

The trend of parameter factor level changes is shown in Fig 7. From the figure, it can be seen that the algorithm performs best when Popsize = 100, Iteration = 1000, and r = 100.

Table 5 $\text{L}_{16}(3^{3})$-orthogonal experiment table and results

Fig 7. Algorithm factor level change curve

*5) Computational complexity is an important factor in any optimization algorithm and it is suggested that the authors add the computational complexity of the proposed IBBPSO and original algorithms and compare the differences.*

**Response:** Thank you for your insightful suggestion. In response to your comment, we conducted a comparative analysis of the computational complexity of the proposed IBBPSO algorithm and the original PSO algorithm. The results are presented in Table 8. Please see the red font in Section 5.3.2 on page 23 of the revised manuscript. The details are as follows :

Table 8 is for the time complexity analysis of the IBBPSO algorithm and PSO algorithm. The runtime results show that the average runtime of the IBBPSO algorithm is 4.332s, slightly higher than the 3.658s of the PSO algorithm, representing an 18.4% increase. This increase is attributed to the more refined particle position adjustment strategy and enhanced global-local search mechanism in the IBBPSO algorithm, which increase the computational complexity of each iteration. However, the improvements in solution quality and faster convergence speed demonstrate that this trade-off is reasonable, highlighting the superiority and stability of the IBBPSO algorithm.

Table 8 Comparison of runtime

*6) Indicate whether uncertainty analysis or other forms of error analysis were performed*

**Response:** Thank you for your valuable suggestion. In response to your comment, we have conducted an error analysis to assess the performance and stability of the proposed IBBPSO algorithm, compared to other algorithms. Specifically, we used **box plots** to statistically analyze the results from 10 runs for each algorithm, as shown in Fig. 10. The box plots visually display the distribution of the results and allow for a comparison of the error spread among different algorithms. Please see the red font in Section 5.3.2 on pages 22-23 of the revised manuscript. The details are as follows :

To compare the result errors of different algorithms in solving this model, box plots were used to statistically analyze the results of 10 runs for each algorithm, as shown in Fig 10. The data distribution indicates that the median of the IBBPSO algorithm is closest to the mean and significantly higher than those of other algorithms, suggesting that this algorithm exhibits greater stability and minimal error.

**Fig 10. Box plots of different algorithms**

*7)* *Discuss how the proposed method addresses partial observability and the curse of dimensionality. Elaborate on strategies employed to mitigate these issues.*

**Response:** Thank you very much for your constructive suggestions. Regarding the issue of **partial observability**, this paper establishes a **comprehensive mathematical model** that encompasses all key **decision variables** and **constraints** within the system, ensuring full observability of the problem. As for the **curse of dimensionality**, the selected **decision variables** in this paper are relatively small in scale, and since the problem addressed by the **IBBPSO algorithm** is a **continuous optimization problem**, the impact of the **curse of dimensionality** is minimal.

*8) Provide more comparisons between the proposed IBBPSO and other meta-heuristic algorithms. This will help confirm the superiority of the proposed method.*

**Response:** Thank you for your valuable suggestion. In response to your comment, we have further strengthened the comparison between the proposed IBBPSO algorithm and other meta-heuristic algorithms. Specifically, we added additional comparisons with the **GWO (Grey Wolf Optimizer) and WOA (** **Whale Optimization Algorithm)** algorithm, which is known for its efficiency in solving multi-objective optimization problems. These comparisons are presented in Table 7,Fig 8 and Fig 9. Please see the red font in Section 5.3.1 on page 21 and Section 5.3.2 on pages 21-22 of the revised manuscript. The details are as follows :

Table 7. Numerical results under different algorithms

The numerical results in Table 7 highlight the superior performance of the proposed IBBPSO algorithm compared to other algorithms. Specifically, the IBBPSO algorithm achieves the highest comprehensive benefits $\text{V}$of 376.447, which is 11.09% and 9.8% higher than the BBPSO and PSO algorithms, respectively. Compared to the GWO and WOA algorithms, the comprehensive benefits V of the IBBPSO algorithm are 33.57% and 17.7% higher, respectively, demonstrating its excellent optimization capability across multiple objectives. In terms of flexibility benefits$\text{V}_{\text{F}}$, the IBBPSO algorithm achieves a value of 296.229, which is 14.79% and 9.21% higher than BBPSO and PSO, respectively, and 41.87% and 54.74% higher than GWO and WOA, respectively. Furthermore, the IBBPSO algorithm demonstrates significant advantages in renewable energy consumption benefits $\text{V}_{\text{R}}$ and environmental benefits$\text{V}_{\text{CO2}}$, highlighting its effectiveness in balancing economic, environmental, and flexibility objectives.

5.3.2 Algorithm Comparison Analysis

Fig 8 shows the convergence curve comparison diagram of the algorithm—the significant advantages of the IBBPSO algorithm in convergence performance. Compared with the PSO, BBPSO, GWO and WOA, the convergence speed is faster, and the convergence result is better. The ' multi-factor ANOVA ' is used to analyze the comprehensive benefit results with a 95 % confidence interval to compare the influence of different algorithms on solving the optimization model. The results are shown in Fig 9. The comprehensive benefit obtained by the IBBPSO algorithm is significantly higher than that of the other two algorithms, which proves its superior optimization ability.

Fig 8. Algorithm convergence curve

Fig 9. Comparison of system benefits of different algorithms

*9) The paper lacks a robust evaluation of the proposed model. Benchmark your results against previous works to demonstrate the advantages and effectiveness of the methodology.*

**Response:** Thank you for your valuable suggestion. In response to your comment, we have conducted a more robust evaluation of the proposed IBBPSO algorithm by benchmarking it against several widely used test functions, as shown in Table 9. Please see the red font in Section 5.3.3 on pages 23-24 of the revised manuscript. The details are as follows :

**5.3.3 Qualitative analysis of different algorithms**

To evaluate the stability of the IBBPSO algorithm, benchmark comparisons are conducted using the Hartmann 4-D, Schaffer F7, and Kowalik and Osborne test functions. The expressions of the benchmark functions and their associated parameters are shown in the Table 9, with a population size of Popsize = 50 and a maximum number of iterations Iteration = 500. The corresponding function plots and simulation results are presented in Figs 10–12. The convergence curves of different algorithms demonstrate that the IBBPSO algorithm exhibits significantly superior convergence speed and final results compared to the other algorithms.

**Table 9 Benchmark function**

(a) Hartmann 4-D three-dimensional function image (b) Algorithm convergence curve comparison

**Fig 10. Image and optimization comparison diagram for the Hartmann 4-D function**

(a) Schaffer F7 three-dimensional function image (b) Algorithm convergence curve comparison

**Fig 11. Image and optimization comparison diagram for the Schaffer F7 function**

(a) Kowalik and Osborne three-dimensional function image (b) Algorithm convergence curve comparison

**Fig 12. Image and optimization comparison diagram for the Kowalik and Osborne function**

*10) Clearly explain the limitations of this work and outline potential directions for future research.*

**Response:** Thank you for your valuable comment. In response to your suggestion, we have revised the manuscript to clearly explain the limitations of this study and outline potential directions for future research. Please see the red font in Conclusion on page 28 of the revised manuscript. The details are as follows :

The limitations of this study primarily lie in the insufficient representativeness of the data, simplified model assumptions, and the computational efficiency of the algorithm, which may affect its applicability in different regions and larger-scale systems. Future research could enhance flexibility evaluation by expanding data sources, integrating various types of information, implementing dynamic assessment and real-time optimization, and introducing multi-objective optimization and intelligent scheduling methods such as reinforcement learning. These advancements would improve the accuracy and timeliness of flexibility assessments, better address the complex and changing demands of power systems, and promote efficient and sustainable operation of power grids.
